# Housing temperature influences exercise‐induced glucose regulation and expression of exerkines in mice

**DOI:** 10.1113/EP092319

**Published:** 2024-12-25

**Authors:** Zhijian Rao, Xue Geng, Peng Huang, Qiangman Wei, Shijie Liu, Chaoyi Qu, Jiexiu Zhao

**Affiliations:** ^1^ Exercise Biological Center China Institute of Sport Science Beijing China; ^2^ College of Physical Education Shanghai Normal University Shanghai China; ^3^ College of Physical Education Hebei Normal University Hebei China

**Keywords:** exercise, exerkine, housing temperature, metabolic health, thermoneutral

## Abstract

The impact of housing temperature on exercise‐induced metabolic adaptations is not well understood, despite extensive research on the benefits of exercise for metabolic health. The aim of this study was to elucidate how housing temperatures influence the molecular responses and metabolic benefits of exercise in mice. Male C57BL/6N mice were housed at either room temperature (RT, 21°C) or in a thermoneutral environment (TN, 29°C) and subjected to either a 6‐week or acute exercise regimen. The results demonstrated that chronic exercise in TN conditions significantly improved glucose tolerance, whereas no such improvement was observed in RT conditions. Exercise reduced adipocyte size in inguinal and epididymal white adipose tissue in RT conditions, but no significant exercise‐induced browning of inguinal white adipose tissue was detected at either housing temperature. Additionally, housing temperature predominantly influenced key metabolic proteins in skeletal muscle, with exercise and temperature exhibiting interactive effects on glycogen synthase, Glut4 and Pgc‐1α. Moreover, the regulation of exerkines, including Fgf21, fetuin‐A, irisin, Gdf15, spexin and apelin, was temperature dependent after both long‐term and acute exercise. Notably, expression of Metrnl was consistently upregulated in skeletal muscle after long‐term exercise in both RT and TN environments, but was downregulated after acute exercise. These findings highlight that environmental temperature critically modulates the metabolic benefits of exercise and the expression of exerkines. The results of this study suggest that conventional RT conditions might obscure the full metabolic effects of exercise. We recommend the use of TN conditions in future research to reduce confounding factors and provide a more accurate assessment of the metabolic benefits of exercise.

## Abstract (highlights)

- **What is the central question of this study?**
  How does housing temperature (room temperature vs. thermoneutral) influence the metabolic adaptations and molecular responses to exercise in mice?
- **What is the main finding and its importance?**
  We found that thermoneutral housing enhances glucose tolerance and differentially regulates key metabolic proteins and exerkine expression during exercise compared with room temperature. This suggests that standard room temperature conditions might obscure the full metabolic benefits of exercise, underscoring the need for thermoneutral conditions in future studies for clearer insights into the metabolic impacts of exercise.

## 1 INTRODUCTION

With the global rise in metabolic diseases, such as diabetes and obesity (Chew et al., 2023), finding effective prevention and treatment strategies has become a crucial task in modern medicine and health sciences. Exercise, as a non‐pharmacological intervention, is widely recognized for its significant role in improving metabolic health and reducing disease risk (Thyfault & Bergouignan, 2020). Exercise is also known to improve glucose tolerance, an essential factor in metabolic health (Young et al., 1989; Zanuso et al., 2010). However, despite the extensive research confirming the health benefits of exercise, our understanding of the molecular mechanisms by which exercise regulates metabolism remains limited. Understanding the specific molecular responses that contribute to improved metabolic health is crucial for developing targeted exercise interventions for metabolic diseases.

One possible mechanism involves the role of exerkines in regulating metabolic health (Turkel et al., 2022). Exercise modulates the expression of various exerkines in muscle, adipose tissue and liver, such as Fgf21, irisin and Metrnl. These exerkines promote glucose and lipid metabolism, exhibit anti‐inflammatory effects and contribute to overall health benefits (Chow et al., 2022). They not only affect the metabolic activity of tissues but also act as endocrine signals between organs, facilitating systemic metabolic coordination and regulation (Walzik et al., 2024).

Another mechanism by which exercise improves metabolic health is through the browning of white adipose tissue. This process, characterized by the transformation of white adipose tissue [such as inguinal white adipose tissue (iWAT)] into brown adipose‐like cells, is marked by the upregulation of key genes, such as *Ucp1*, *Prdm16*, *Cidea* and *Pgc‐1α* (Chand et al., 2024; Machado et al., 2022). Browning enhances the capacity for energy expenditure of adipose tissue, which can significantly influence whole‐body energy metabolism and weight regulation (Ghesmati et al., 2024; Zhu et al., 2024). In room temperature (RT) environments, exercise has been shown to induce this transformation, thereby potentially contributing to improved metabolic health (Di et al., 2020; Tanimura et al., 2022; Wang et al., 2022).

However, most current research is based on mouse models, and standard laboratory housing temperatures (∼21°C) are below the thermoneutral (TN) temperature for mice (∼30°C). In this mildly cold environment, the physiological activities of mice, including cardiovascular, metabolic and immune functions, are significantly affected (Maloney et al., 2014). Mice, owing to their higher surface‐to‐volume ratio, are more sensitive to their thermal environment, whereas humans can adapt to sub‐TN temperatures more easily through behavioural modifications, such as wearing clothing. The influence of environmental temperatures on the metabolic effects of exercise, particularly in the context of TN and sub‐TN conditions, remains an area that requires further investigation. Recent studies have explored how housing temperature influences exercise adaptations in mice, highlighting the challenges of extrapolating research findings in mice to humans. For instance, one study (Raun et al., 2020) found that at lower temperatures (RT), mice have a higher basal metabolic rate owing to the additional energy required to maintain body temperature. Consequently, exercise training at low temperatures might lead to more pronounced metabolic and physiological adaptations, such as greater browning of fat and changes in metabolic gene expression. In contrast, at TN temperatures, the effects of exercise training are less pronounced owing to the lower basal metabolic rate and energy demands. Another study investigated the effects of environmental temperature on acute and chronic metabolic responses to exercise (McKie et al., 2019). It found that acute exercise at lower temperatures might lead to more significant metabolic changes, whereas chronic exercise adaptations are less evident at TN temperatures.

The aim of this study was to explore the impact of housing temperature on the metabolic regulation of exercise, specifically focusing on the molecular responses of mice to exercise in RT and TN environments. We analysed how different housing temperatures affect metabolic health and the expression of exerkines in mice, to gain a deeper understanding of the role of housing temperature in the metabolic regulation of exercise. This research will help to elucidate the mechanisms by which exercise improves metabolic health, contributing to more effective exercise‐based interventions for preventing and treating metabolic diseases.

## 2 MATERIALS AND METHODS

### 2.1 Ethical approval

This study achieved ethical approval by the Ethics Committee of the China Institute of Sport Science (CISSLA‐20230217). All animal experiments were conducted in accordance with the guidelines of the Institutional Animal Care and Use Committee.

### 2.2 Experimental animals and grouping

A total of 72 8‐week‐old male C57BL/6N mice were sourced from Beijing Vital River Laboratory Animal Technology Co., Ltd [SCXK (Jing) 2021‐0006]. Male mice were housed in standard cages (three mice per cage) in a controlled environment with a 12 h light–12 h dark cycle. The relative humidity in the housing environment was maintained between 40% and 70% throughout the experiment. All mice were fed a standard chow diet (providing 10% kcal from fat, 20% kcal from protein and 70% kcal from carbohydrates) throughout the study. Water was provided ad libitum. Thirty‐six mice were randomly assigned to the RT group and another 36 to the TN group. The experimental design included acute and chronic exercise interventions. For the acute exercise part, mice acclimated to their respective housing environments for 4 weeks before being randomly assigned to one of four groups: thermoneutral control (TN‐Con), thermoneutral acute exercise (TN‐AE), room temperature control (RT‐Con) and room temperature acute exercise (RT‐AE), with nine mice per group. For the chronic exercise part, mice acclimated for 2 weeks, then were randomly assigned to thermoneutral sedentary (TN‐Sed), thermoneutral exercise training (TN‐ET), room temperature sedentary (RT‐Sed) and room temperature exercise training (RT‐ET) groups, with each group consisting of nine mice. Housing temperatures were set based on literature (Aldiss et al., 2020; McKie et al., 2019; Raun et al., 2020), with TN conditions at 29°C and RT conditions at 21°C.

### 2.3 Measurement of food intake

Each morning, 100 g of chow diet was placed in each cage. The remaining food was weighed 24 h later to determine the amount consumed. The total food intake for each cage was divided by the number of mice (three per cage) to calculate the daily food intake per mouse. The daily intake values were averaged over the 7‐day period to obtain the average daily food intake per mouse for that week.

### 2.4 Exercise protocol

Before exercise interventions, mice designated for both acute and chronic exercise experiments underwent a 3‐day adaptation training. The training protocol was consistent for both experiments and included the following. On the first day, the speed started at 6 m/min (treadmill incline 0°), with a 2 m/min increment every 2 min until reaching 12 m/min, totalling 15 min of exercise. On the second and third days, the duration remained constant, but maximal speeds increased to 14 and 16 m/min, respectively. For acute exercise, mice had a day of rest after the adaptation training before undergoing a single session of moderate‐intensity exercise, lasting 60 min, following a previously established protocol (Landry et al., 2021). For chronic exercise, mice exercised once daily, 5 days a week, for a total of 6 weeks (30 days). A progressive load programme was followed, with intensity and duration increasing weekly and per session, respectively. Intensity started at 12 m/min and increased by 1 m/min weekly until reaching 15 m/min, which then remained constant. Exercise duration began at 31 min, increasing by 1 min after each session, reaching 60 min at the last exercise session. The exercise environment was consistent with the housing environment.

### 2.5 Glucose tolerance test

The glucose tolerance test was conducted in the same respective housing environments (TN or RT), using a glucose solution at a dosage of 2 g/kg. Glucose was dissolved in saline to a concentration of 20%. Mice were fasted for 16 h with free access to water prior to the test. Baseline fasting blood glucose was measured using tail vein blood collection, recorded as the 0 min value. Subsequently, glucose was administered via intraperitoneal injection, with a dosage of 0.01 mL/g body weight (BW). Blood glucose levels were measured at 15, 30, 60, 90 and 120 min after glucose administration using a glucometer.

### 2.6 Sample collection

For the acute exercise part, after a single exercise session the mice were killed immediately by cervical dislocation. The liver, iWAT, epididymal white adipose tissue (eWAT) and anterior tibialis (TA) muscles were swiftly exercised, weighed, flash‐frozen in liquid nitrogen, and stored at −80°C. For the chronic exercise part, mice were killed 24 h after their last exercise session using the same procedure, and samples were handled in a similar manner. Before the end of the experiment, the mice were fasted for 12 h to standardize metabolic conditions.

### 2.7 Real‐time quantitative PCR

Approximately 20 mg of tissue, including adipose tissue, TA muscle and liver, was homogenized in 200 µL of Trizol reagent, with total RNA subsequently extracted according to the manufacturer's instructions. The extracted RNA was reverse transcribed into complementary DNA using the Novozyme HiScript III All‐in‐one RT SuperMix kit (R333), and real‐time quantitative PCR experiments were conducted following the Taq Pro Universal SYBR qPCR Master Mix kit (Q712) instructions. The primer sequences used in this study are provided in Table 1.

**TABLE 1:** Primer sequences.

Gene	Forward	Reverse
*Actb*	ATCACTATTGGCAACGAGCGGTTC	CAGCACTGTGTTGGCATAGAGGTC
*Adipolin*	GGATTCCAAGCTCCTACTACTC	AGATGAGAACACGGACCATATC
*Spexin*	TCCTTCTCCTGGTGCTGTCTG	GCTCCTTCCTACGGCTCTGG
*Adiponectin*	CCAATGTACCCATTCGCTTTAC	GAAGTAGTAGAGTCCCGGAATG
*Leptin*	ATAGCCAATGACCTGGAGAATC	CCAACTGTTGAAGAATGTCCTG
*Fetuin‐A*	GACTTTCAGACCACCGAACTTA	AGAATGAAGAGTTTCTCCCGAG
*Apelin*	AACAGGACTAGAAGAAGGAAGC	CTGTCTGCGAAATTTCCTCCT
*Metrnl*	GCTGCTGTTGCTGCTACTACTG	TCCTTGCTGCGTGCCTCTC
*Gdf15*	ATACTCAGTCCAGAGGTGAGAT	CTTCAGGGGCCTAGTGATG
*Irisin*	AAGGACAACGAGCCCAATAACAAC	TCATATCTTGCTGCGGAGGAGAC
*Fgf21*	CGGTTACAATGTGTACCAGTCT	GTAAAGGCTCTACCATGCTCAG
*Prdm16*	CAACAAAGAGAAGCCGTTCAAG	TTTCGGATCTCGGAGAAGTAAG
*Ucp1*	ATTCAGAGGCAAATCAGCTTTG	GTGTTTCTCTCCCTGAAGAGAA
*Cidea*	CAATGTCAAAGCCACGATGTAC	CTGTGCAGCATAGGACATAAAC
*Pgc‐1a*	GGATATACTTTACGCAGGTCGA	CGTCTGAGTTGGTATCTAGGTC

### 2.8 Western blotting

Tissue was homogenized in cell lysis buffer supplemented with phenylmethylsulphonyl fluoride and protease inhibitor cocktail. Protein quantification was conducted using a bicinconinic acid assay. Subsequently, samples were loaded onto 15‐well acrylamide gels, transferred to nitrocellulose membranes, and incubated overnight at 4°C with primary antibodies sourced from various vendors. These included AbCam (ON, USA) for UCP1 (ab209483), GS (ab40810) and p‐GS (ab81230); Proteintech (Wuhan, China) for PGC‐1α (66369‐1‐Ig), FIS1 (10956‐1‐AP), MFN2 (12186‐1‐AP), GLUT4 (66846‐1‐Ig), CPT‐1α (15184‐1‐AP), ACTIN (81115‐1‐RR) and GAPDH (60004‐1‐Ig); and Abclonal (Wuhan, China) for PDK4 (A13337) and COQ10b (A15193).

### 2.9 Histology

Histology was completed as previously described (Rao et al., 2018).

### 2.10 Measurement of adipocyte size

Adipocyte size was measured using Haematoxylin‐ and Eosin‐stained sections of the iWAT and eWAT. Whole tissue sections were scanned and multiple non‐overlapping fields randomly selected for analysis. The images were captured at ×20 magnification, and adipocyte area was quantified using ImageJ software (National Institutes of Health, Bethesda, MD, USA). At least 200 adipocytes per animal were measured to ensure reliable size distribution data. The results were expressed as the average adipocyte cross‐sectional area.

### 2.11 Data analysis and statistical methods

All experimental data were analysed using SPSS software with a two‐way ANOVA to assess the presence of main effects or interaction effects. If the analysis indicated significant main or interaction effects, Tukey's *post hoc* tests were performed to identify specific group differences. Graphs were generated using GraphPad Prism v.9.0, and all results are presented as the mean ± SD. The statistical significance level for intergroup differences was set at *P *< 0.05.

## 3 RESULTS

### 3.1 Effects of chronic exercise on body weight, tissue mass and glucose tolerance in mice in different temperature conditions

**FIGURE 1:**
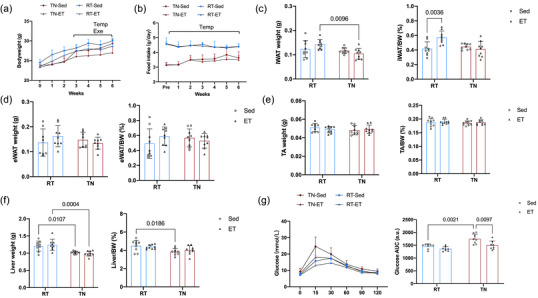
The effects of chronic exercise on body weight, tissue mass and glucose tolerance in mice in different temperature conditions. (a) Mice experienced an increase in body weight throughout the intervention period. From week 3, both exercise and temperature exerted significant main effects on body weight. (b) Mice housed at RT consumed significantly more food than those in the TN environment. (c) Chronic exercise did not significantly alter iWAT mass in either environment. However, in the RT environment, the relative iWAT weight increased significantly after chronic exercise, whereas no changes were observed in the TN environment. (d) There were no significant changes in eWAT mass or relative weight ratio in any group or environment. (e) There were no significant differences in TA mass or relative weight ratio observed across conditions. (f) Liver mass was significantly higher in the RT environment, but the relative liver mass was significantly increased only in the sedentary group at RT compared with the TN sedentary group. (g) In the glucose tolerance test, sedentary mice in the RT environment displayed significantly better glucose tolerance than those in the TN environment. Chronic exercise improved glucose tolerance significantly in the TN environment, but there were no differences between the sedentary and chronic exercise groups in the RT environment. Data (*n* = 9 mice per group) are presented as the mean ± SD, including individual values where applicable. Abbreviations: ET, exercise training; eWAT, epididymal white adipose tissue; iWAT, inguinal white adipose tissue; RT, room temperature; Sed, sedentary; TA, tibialis anterior; Temp, main effect of temperature; Temp*Exe, exercise by temperature interaction; TN, thermoneutral.

During the intervention, BW increased in all groups, with significant main effects of both chronic exercise and temperature starting from the third week (Figure 1a). Food intake showed a significant temperature effect, with mice in the RT conditions consuming significantly more than those in the TN environment (Figure 1b). We analysed data from mice that underwent chronic exercise, and results showed that chronic exercise had no significant effect on iWAT mass in either condition, but the relative iWAT mass (iWAT/BW ratio) increased significantly in the chronic exercise group in RT conditions, whereas no significant change was observed in the TN environment (Figure 1c). No significant changes were observed in eWAT mass or its relative weight ratio across both conditions and exercise interventions (Figure 1d). Likewise, TA mass and its relative ratio did not show significant differences between any groups (Figure 1e). Liver mass was significantly greater in the RT group compared with the TN group, but the relative liver weight was significantly elevated only in the RT sedentary group compared with the TN sedentary group (Figure 1f). The glucose tolerance test revealed that the RT sedentary group had significantly better glucose tolerance than the TN sedentary group. Chronic exercise improved glucose tolerance significantly in the TN environment, whereas no significant difference was seen between the chronic exercise and sedentary groups at RT (Figure 1g).

### 3.2 Differential adipocyte response to chronic exercise in different temperature conditions

**FIGURE 2:**
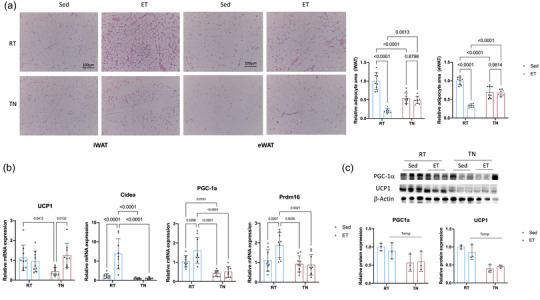
Temperature‐dependent effects of chronic exercise on tissue morphology, adipocyte size, gene expression and protein levels. (a) Representative histological images (Haematoxylin and Eosin staining) of iWAT and eWAT from sedentary and chronic exercise groups in RT and TN conditions (*n* = 9). In RT conditions, exercise led to darker staining of adipose tissue, suggesting metabolic changes, although no multilocular adipocytes were observed. In the TN environment, this staining effect was suppressed. Adipocyte size was significantly reduced in the chronic exercise group compared with the sedentary group at RT, whereas no significant difference was observed between groups in the TN environment. Additionally, adipocyte size in the TN sedentary group was significantly smaller than that in the RT sedentary group. (b) Relative mRNA expression levels of *Ucp1*, *Pgc‐1α*, *Prdm16* and *Cidea* in iWAT (*n* = 9). In RT conditions, chronic exercise upregulated *Pgc‐1α*, *Prdm16* and *Cidea*, but *Ucp1* expression remained unchanged. In TN conditions, *Ucp1* was significantly upregulated after chronic exercise, whereas *Pgc‐1α*, *Prdm16* and *Cidea* showed no significant changes. (c) Western blot analysis of Ucp1 and Pgc‐1α protein levels in iWAT from sedentary and chronic exercise groups in both RT and TN conditions (*n* = 3). No significant differences in protein levels were observed between the chronic exercise and sedentary groups in either condition. However, a temperature effect was observed, with significantly higher expression of both proteins in RT compared with TN. Data are presented as mean ± SD, including individual values where applicable. Temp, main effect of temperature. Abbreviations: ET, exercise training; eWAT, epididymal white adipose tissue; iWAT, inguinal white adipose tissue; RT, room temperature; Sed, sedentary; Temp, main effect of temperature; TN, thermoneutral.

In the RT environment, for animals that underwent exercise training, we observed that chronic exercise reduced adipocyte size compared with the sedentary group, whereas in the TN environment there was no significant difference in adipocyte size between the two groups (Figure 2a). Interestingly, in the TN environment, the adipocyte size of the sedentary group was significantly smaller than that of the sedentary group in the RT environment (Figure 2a). Additionally, after chronic exercise in the RT environment, adipose tissue staining became darker, although no multilocular adipocytes were observed, whereas this staining effect was suppressed in the TN environment.

Gene expression results showed that chronic exercise in the RT environment led to the upregulation of *Pgc‐1α*, *Prdm16* and *Cidea* but did not significantly affect *Ucp1* expression (Figure 2b). Conversely, in the TN environment, *Ucp1* expression was significantly upregulated, but there were no significant changes in *Pgc‐1α*, *Prdm16* or *Cidea* (Figure 2b).

At the protein level, analysing samples from chronically exercised animals, we found that there were no significant differences in Ucp1 and Pgc‐1α between the chronic exercise and sedentary groups in either environment (Figure 2c). However, there was a temperature effect, with significantly higher expression of both proteins in the RT environment compared with the TN environment (Figure 2c).

### 3.3 Effects of chronic exercise and housing temperature on key metabolic proteins in TA

**FIGURE 3:**
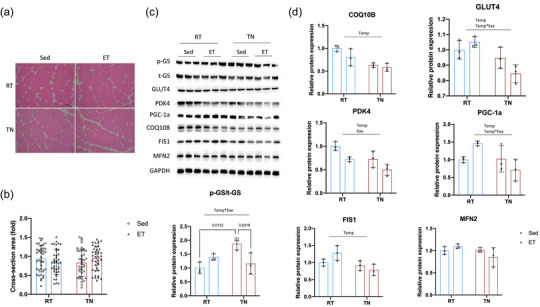
Morphology and molecular analysis of TA muscle across temperature and chronic exercise conditions. (a, b) Representative images of TA morphology, showing no significant differences between groups. (c) Protein expression levels of p‐GS (phosphorylated glycogen synthase), t‐GS (total glycogen synthase), GLUT4, PDK4, PGC‐1α, COQ10B, FIS1 and MFN2 were analysed via western blot. (d) Quantification of protein expression levels. A significant main effect of temperature was observed for GLUT4, PDK4, PGC‐1α, COQ10B and FIS1, indicating that their expression levels were higher in one temperature condition compared with the other. Chronic exercise had no significant effect on these proteins in either temperature condition. Additionally, the p‐GS/t‐GS ratio showed a significant interaction between exercise and temperature. In TN, chronic exercise significantly reduced the p‐GS/t‐GS ratio compared with the sedentary group, whereas no such effect was seen in RT. Data (*n* = 3 mice per group) are presented as the mean ± SD, including individual values where applicable. Abbreviations: ET, exercise training; Exe, main effect of chronic exercise; RT, room temperature; Sed, sedentary; TA, tibialis anterior; Temp, main effect of temperature; Temp*Exe, exercise by temperature interaction; TN, thermoneutral.

In skeletal muscle, increasing glucose uptake and utilization and enhancing mitochondrial function and oxidative capacity are potential mechanisms by which exercise improves glucose tolerance (Esteves & Stanford, 2024; Smith et al., 2023). We found no significant differences in TA morphology across groups (Figure 3a, b). Next, the expression levels of p‐GS, t‐GS, Glut4, Pdk4, Pgc‐1α, Coq10b, Fis1 and Mfn2 in the TA were analysed (Figure 3c, d). Glut4, Pdk4, Pgc‐1α, Coq10b and Fis1 showed a significant main effect of temperature, indicating that their expression levels varied significantly between different temperature conditions, whereas chronic exercise did not significantly affect their expression in either temperature condition. This might explain, in part, why glucose tolerance is better in sedentary mice housed at RT compared with those in a TN environment.

The p‐GS/t‐GS ratio exhibited an interaction effect between chronic exercise and temperature, with the TN environment showing a significantly higher p‐GS/t‐GS ratio than the RT environment. In the RT environment, chronic exercise did not significantly affect the p‐GS/t‐GS ratio, whereas in the TN environment, chronic exercise significantly reduced the p‐GS/t‐GS ratio compared with the sedentary group. These results suggest that exercise in the TN environment significantly reduced the level of phosphorylated glycogen synthase (p‐GS), indicating increased GS activity, which could enhance glycogen synthesis and storage, thereby improving glucose utilization and tolerance in the muscle.

### 3.4 Temperature‐dependent modulation of expression of exerkines by long‐term exercise

**FIGURE 4:**
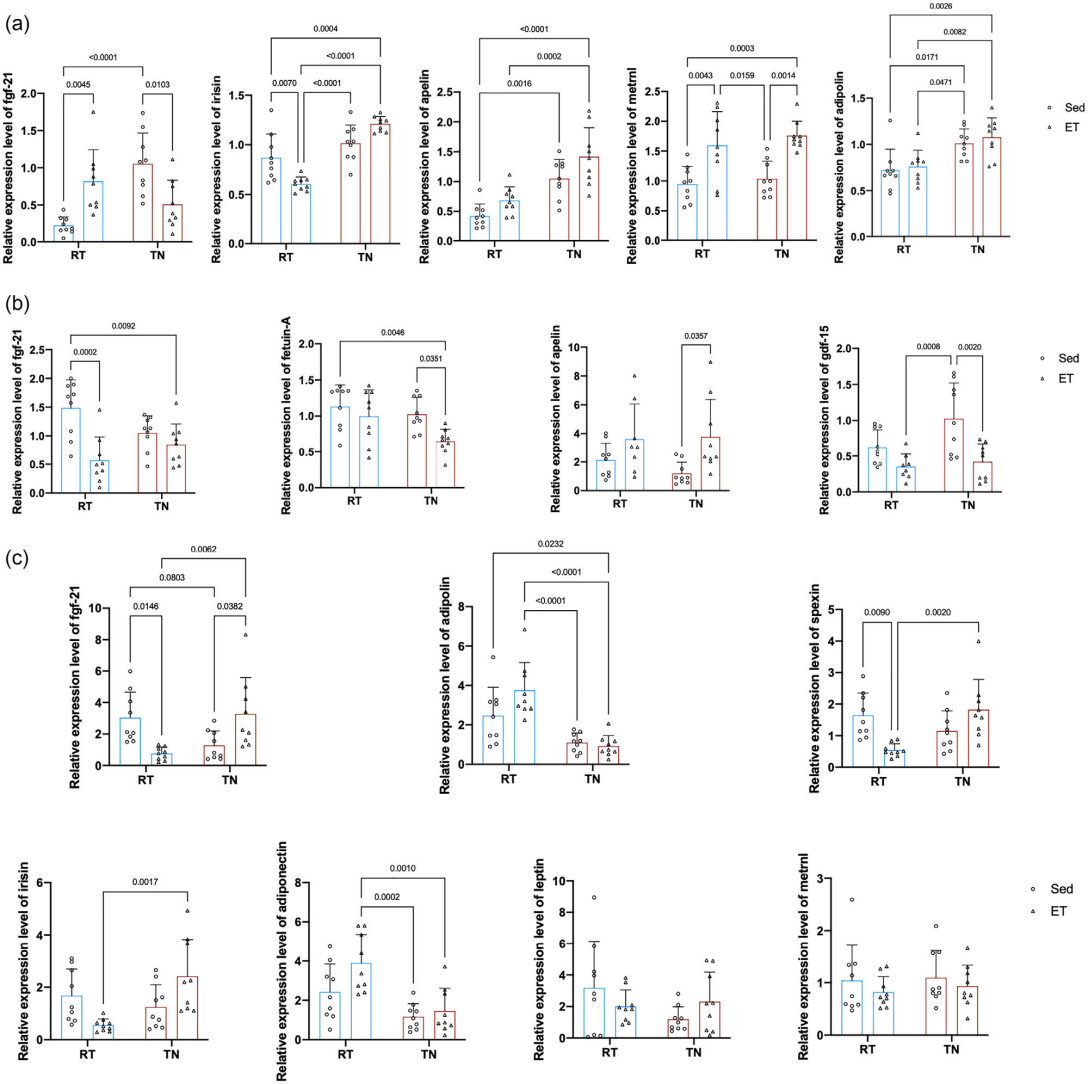
Temperature‐dependent modulation of exerkine expression by long‐term exercise. (a) Expression levels of exerkines (*Fgf21, Irisin, Apelin, Metrnl and Adipolin*) in TA muscle with different housing temperatures. (b) Expression levels of exerkines (*Fgf21, Fetuin‐A, Apelin and Gdf15*) in the liver with different housing temperatures. (c) Expression levels of exerkines (*Fgf21, Adipolin, Spexin, Adiponectin, Irisin, leptin and Metrnl*) in inguinal white adipose tissue with different housing temperatures. Data (*n* = 9 mice per group) are presented as the mean ± SD, including individual values where applicable. Abbreviations: ET, exercise training; RT, room temperature; Sed, sedentary; TA, tibialis anterior; TN, thermoneutral.

Exerkines are signalling molecules released by tissues in response to physical activity, playing crucial roles in metabolic regulation and adaptation (Chow et al., 2022). Exercise can regulate body metabolism through the modulation of exerkines. We investigated the effects of long‐term exercise on the expression of exerkines in the TA (Figure 4a), liver (Figure 4b) and iWAT (Figure 4c) in different housing temperatures. Our results demonstrated that in a TN environment, long‐term exercise decreased the expression of *Fgf21* but increased *Metrnl* in skeletal muscle, decreased *Gdf15* and *Fetuin‐A*, and increased *Apelin* in the liver, and upregulated *Fgf21* in iWAT. Conversely, in the RT environment, long‐term exercise elevated *Fgf21* and *Metrnl* but reduced *Irisin* in skeletal muscle, downregulated *Fgf21* in the liver, and decreased *Fgf21* and *Spexin* in iWAT. These findings suggest that the regulatory effects of exercise on expression of exerkines are influenced by ambient temperature. The upregulation of *Metrnl* in muscle at both housing temperatures indicates its robust response to exercise. The differential expression of liver and iWAT exerkines highlights the complex interplay between housing temperatures and metabolic adaptations. In summary, these results reveal that housing temperature modulates the expression of exerkines, which might underlie the varied metabolic benefits observed with exercise in different environmental conditions.

### 3.5 Impact of acute exercise on expression of exerkines at different housing temperatures

**FIGURE 5:**
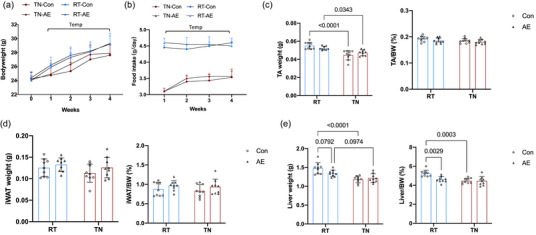
Impact of AE on TA muscle, iWAT and liver in different housing temperatures. (a) Changes in BW after 4 weeks of acclimation to RT and TN environments. (b) Food intake during the acclimation period. (c) The TA/BW ratio. (d) The iWAT/BW ratio. (e) The liver/BW ratio. Data (*n* = 9 mice per group) are presented as the mean ± SD, including individual values where applicable. Abbreviations: AE, acute exercise; BW, body weight; Con, control; iWAT, inguinal white adipose tissue; RT, room temperature; TA, tibialis anterior; Temp, main effect of temperature; TN, thermoneutral.

Investigating the effects of acute exercise on exerkine expression is essential for understanding the immediate metabolic responses to physical activity. In this study, mice were acclimated to RT or TN environments for 4 weeks before undergoing acute exercise. During the acclimation period, BW (Figure 5a) and food intake (Figure 5b) showed a significant main effect of housing temperature, but exercise and housing temperature did not significantly affect the TA/BW ratio (Figure 5c) or iWAT/BW ratio (Figure 5d). In the TN environment, acute exercise did not significantly impact the liver/BW ratio (Figure 5e); however, in the RT environment, acute exercise significantly reduced the liver weight index compared with control animals.

**FIGURE 6:**
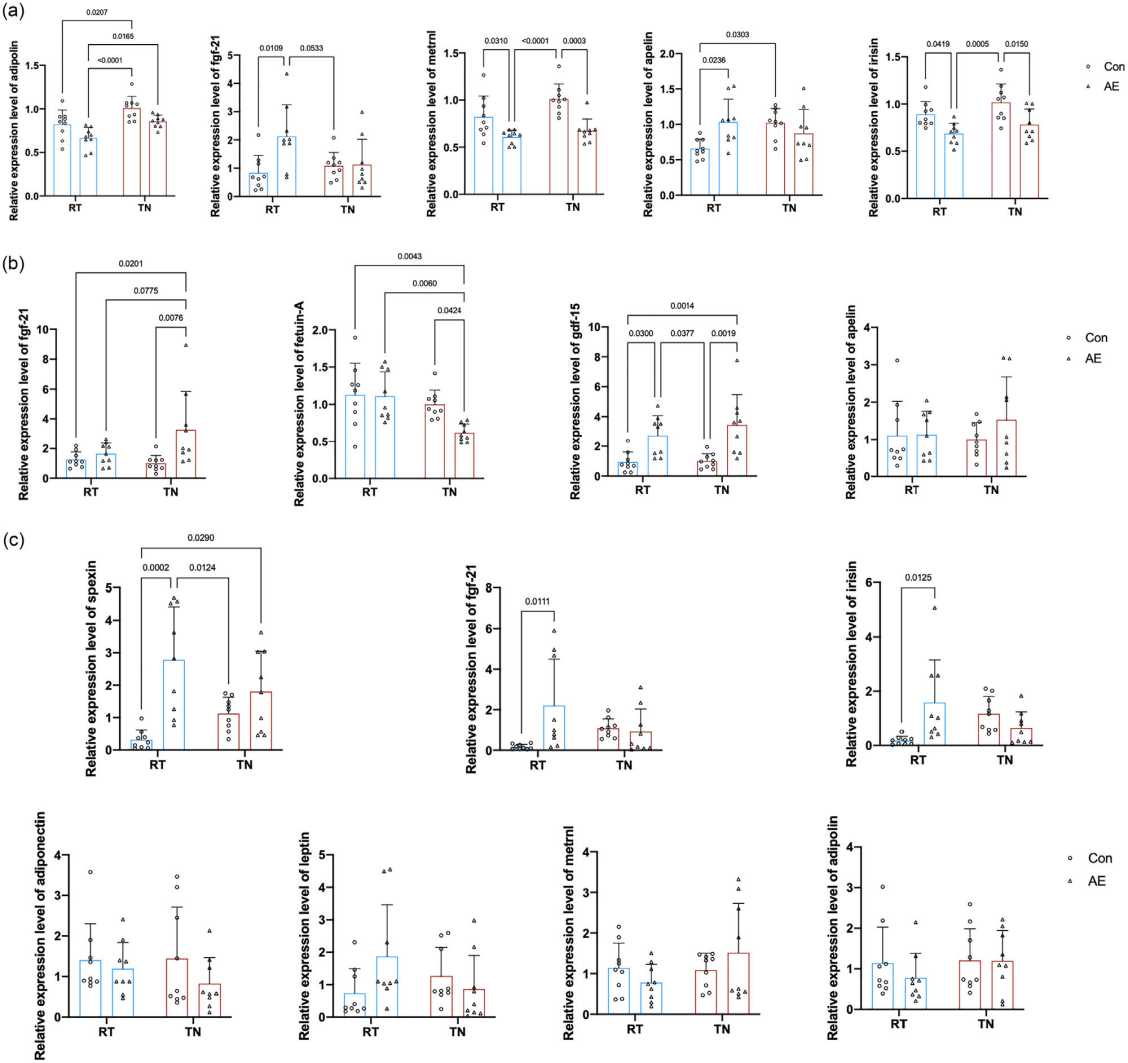
Impact of acute exercise on exerkine expression in different housing temperatures. (a) Expression levels of exerkines (*Fgf21, Irisin, Apelin, Metrnl and Adipolin*) in TA muscle with different housing temperatures. (b) Expression levels of exerkines (*Fgf21, Fetuin‐A, Apelin and Gdf15*) in the liver with different housing temperatures. (c) Expression levels of exerkines (*Fgf21, Adipolin, Spexin, Adiponectin, Irisin, Leptin and Metrnl*) in inguinal white adipose tissue with different housing temperatures. Data (*n* = 9 mice per group) are presented as the mean ± SD, including individual values where applicable. Abbreviations: AE, acute exercise; Con, control; ET, exercise training; RT, room temperature; TA, tibialis anterior; TN, thermoneutral.

Additionally, the liver weight index of control mice in the TN environment was significantly lower than that of control mice in the RT environment. The patterns of exerkine changes in TA (Figure 6a), liver (Figure 6b) and iWAT (Figure 6c) also varied with acute exercise. Specifically, in the TN environment, acute exercise downregulated the expression of *Irisin* and *Metrnl* in skeletal muscle, decreased *Gdf15* and *Fgf21*, and increased *Fetuin‐A* in the liver. In contrast, in the RT environment, acute exercise upregulated *Fgf21* and *Apelin* while downregulating *Irisin* and *Metrnl* in skeletal muscle, increased *Gdf15* in the liver, and upregulated *Fgf21*, *Irisin* and *Spexin* in iWAT. The differential expression of exerkines following acute exercise highlights the importance of considering the housing temperature when evaluating the metabolic benefits of exercise.

**FIGURE 7:**
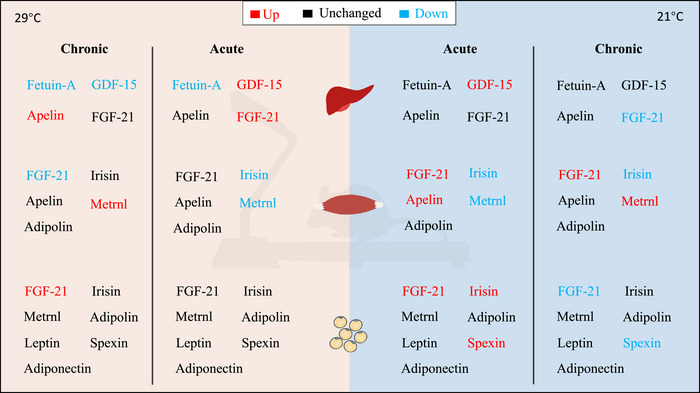
Summary of chronic and acute exercise‐induced exerkine responses modulated by housing temperature.

In summary, chronic and acute exercise induces distinct exerkine responses depending on the housing temperature (Figure 7), suggesting a complex interaction between exercise and housing temperature in regulating metabolic processes.

## 4 DISCUSSION

In the present study, we investigated the impact of housing temperature on the metabolic benefits of exercise, with a focus on glucose tolerance and exerkine expression. Our findings reveal that environmental temperature significantly modulates these physiological responses, suggesting that the commonly used RT in animal studies might confound the interpretation of the true metabolic effects of exercise.

Our results indicate that exercise improves glucose tolerance in a TN environment, but not in an RT environment. This indicates that the metabolic benefits of exercise might be masked, in part, by the stress of cold exposure in RT conditions. Previous literature presents mixed results regarding the effects of exercise and housing temperature on glucose tolerance. One study reported a main effect of voluntary wheel running to improve glucose tolerance and a main effect of temperature suggesting improved glucose tolerance (McKie et al., 2019). Another study found that glucose tolerance improves following voluntary wheel running in mice housed at 22°C, but exercise in TN conditions did not improve glucose tolerance (Raun et al., 2020). These studies were conducted on C57BL/6J mice, which exhibit impaired glucose metabolism owing to a mutation in *Nnt*, characterized by reduced insulin secretion, impaired glucose tolerance and hyperglycaemia (Freeman, Shimomura et al., 2006; Freeman, Hugill et al., 2006). To mitigate this factor, our study used C57BL/6N mice, which do not have this genetic mutation. Despite these precautions, our results were inconsistent with the findings of both previous studies, indicating that the interaction between exercise, housing temperature and glucose metabolism might be more complex and strain specific than previously understood.

To explain why exercise improves glucose tolerance in TN but not RT environments, we examined the effects on the browning of iWAT and skeletal muscle metabolism. Exercise‐induced browning of white adipose tissue has been shown to increase energy expenditure and improve metabolic health (Stanford et al., 2015). The results of the present study indicate that in the RT environment, although exercise reduced the size of adipocytes and histological staining showed a deepening of adipose tissue colour, no multilocular lipid droplets were observed, suggesting that browning had not yet occurred fully. However, the upregulation of genes such as *Pgc‐1α*, *Prdm16* and *Cidea* suggests that early regulatory signals of browning were activated. The upregulation of these genes indicates a trend towards browning in adipose tissue, although *Ucp1* gene and Ucp1 protein levels were not significantly increased, implying that functional browning had not yet developed fully. In contrast, in TN conditions, despite a significant increase in *Ucp1*gene expression following exercise, the key regulatory genes *Pgc‐1α*, *Prdm16* and *Cidea* did not show significant changes, and at the protein level, no significant upregulation of Ucp1 and Pgc‐1α was observed. This suggests that exercise‐induced browning in the TN environment might be inhibited or progress more slowly. Additionally, the suppression of exercise‐induced browning in a TN environment, although exercise still improves glucose tolerance, suggests that the improvement in glucose tolerance attributable to exercise is independent of browning. This aligns with human studies, which often report that exercise does not induce browning of white adipose tissue (Camera et al., 2010; Nakhuda et al., 2016; Pino et al., 2016; Tsiloulis et al., 2018; Zuriaga et al., 2017), yet consistently show that exercise improves glucose tolerance.

In the skeletal muscle, our results indicate that environmental temperature significantly affects the expression levels of *Glut4*, *Pdk4*, *Pgc‐1α*, *Coq10b* and *Fis1*, whereas exercise did not significantly alter the expression of these genes at either temperature. This suggests that environmental temperature influences glucose uptake and metabolism by regulating these genes. Similar findings have been reported by Raun et al. (2020), who noted that environmental temperature significantly impacts the expression of genes related to glucose metabolism. The p‐GS/t‐GS ratio showed an interaction effect between exercise and temperature, with exercise in a TN environment significantly reducing the ratio, indicating increased GS activity, which might enhance glycogen synthesis and glucose utilization. However, exercise did not significantly affect the p‐GS/t‐GS ratio in the RT environment. This suggests that the improvement in glucose tolerance observed in the TN environment might be related to exercise‐induced activation of GS in muscle. Despite this, mice housed in the RT environment exhibited better overall glucose tolerance than those in the TN environment, consistent with previous studies by Kregel et al. (1990), possibly owing to higher expression of *Glut4* and other genes enhancing glucose uptake and utilization. Additionally, the upregulation of mitochondrial fusion protein 2 (Mfn2) in iWAT in the TN environment might also contribute to improved glucose tolerance, as MFN2 is involved in mitochondrial function and metabolic regulation (Emery & Ortiz, 2021; Wang et al., 2023). These findings highlight the crucial role of environmental temperature in regulating glucose tolerance and suggest that studying health benefits of exercise should consider the influence of housing temperature.

Exerkines are crucial in mediating the systemic metabolic effects of exercise (Chow et al., 2022; Jin et al., 2024). To ascertain whether the housing temperature impacts the response of exerkines to exercise, we compared the changes in exerkines after chronic or acute exercise in mice housed at RT or TN.

Gdf15 is expressed in various tissues, including the liver, lungs and kidneys (Fairlie et al., 1999). Human studies have shown that both endurance exercise and resistance training can upregulate circulating levels of GDF15 (Klein et al., 2021; Kleinert et al., 2018). There is limited research on the tissue source contributing to the increased circulating GDF15 after exercise. A recent study reported that GDF15 increased rapidly in response to muscle contraction (Laurens et al., 2020). However, Kleinert et al. (2018) reported that skeletal muscle is not a primary contributor to circulating GDF15 after exercise. Furthermore, Klein et al. (2021) found that *Gdf15* mRNA in the liver of mice increased by approximately twofold after acute exercise. Consistent with these findings, we observed that acute exercise, regardless of whether the mice were housed at RT or TN, can upregulate the expression of *Gdf15* mRNA in the liver. These results suggest that the increase in circulating GDF15 after acute exercise might originate predominantly from the liver.

There is limited research on the impact of chronic exercise on Gdf15 expression. Some human studies suggest an inverse correlation between plasma GDF15 levels and fitness (Conte et al., 2020; Enarsson et al., 2021), indicating that chronic exercise might decrease GDF15 levels. We demonstrated a significant decrease in the expression of *Gdf15* mRNA in the liver of mice after chronic exercise in the TN conditions, whereas there was no significant change in *Gdf15* mRNA expression in the liver of mice after chronic exercise at RT. This suggests that, in terms of the Gdf15 response to exercise, mice housed at TN closely mimic the human physiological response.

Fetuin‐A is expressed primarily in the liver, and it can exacerbate peripheral insulin resistance (Watt et al., 2019). Human studies indicate that plasma fetuin‐A levels do not change significantly after acute exercise (Sargeant et al., 2018) but might decrease with long‐term exercise (Jenkins et al., 2011; Keihanian et al., 2019; Lee et al., 2017; Malin et al., 2014). In our study, acute and long‐term exercise at RT did not significantly affect the expression of fetuin‐A mRNA in the mouse liver. However, both acute and long‐term exercise significantly reduced the expression of liver fetuin‐A mRNA in the mice housed in TN conditions. This suggests that, in terms of fetuin‐A response to exercise, mice housed in TN conditions closely resemble humans in their physiological response.

Fgf21 is expressed in various tissues, including the liver, skeletal muscle and adipose tissue, and it promotes mitochondrial biosynthesis and browning of white adipose tissue (Nishimura et al., 2000). The impact of exercise on Fgf21 expression is complex and influenced by exercise intensity and type and by metabolic status. In general, acute aerobic exercise can increase Fgf21 levels (Kim et al., 2013; Morville et al., 2018; Tanimura et al., 2016), whereas circulating FGF21 levels tend to decrease or remain unchanged after chronic exercise (Kong et al., 2016; Kruse et al., 2017; Scalzo et al., 2014; Taniguchi et al., 2016; Yang et al., 2011).

In our study, acute exercise in the RT condions significantly increased *Fgf21* mRNA expression in TA and iWAT, with no significant change in liver *Fgf21* expression. However, in the TN conditions, acute exercise upregulated *Fgf21* mRNA expression in the liver, whereas no significant changes were observed in TA and iWAT. Considering that acute exercise‐induced circulating FGF21 protein in humans is likely to originate from the liver rather than skeletal muscle (Hansen et al., 2015; Sabaratnam et al., 2018), we argue that concerning the acute response of Fgf21 to exercise, mice housed in TN conditions accurately reflect the human physiological response.

Furthermore, the response of *Fgf21* mRNA to chronic exercise in TN and RT conditions showed almost opposite trends. In the RT conditions, chronic exercise upregulated TA *Fgf21* mRNA and downregulated iWAT *Fgf21* mRNA. In the TN conditions, chronic exercise led to the opposite effect (downregulation of *Fgf21* mRNA expression in TA and upregulation in iWAT). Interestingly, chronic exercise in RT downregulated liver *Fgf21* mRNA expression, whereas no significant change was observed in the TN. These results indicate an interactive influence of housing temperature and chronic exercise on *Fgf21* expression. Considering the regulatory role of cold stimuli on *Fgf21* expression (Hondares et al., 2011; Lee et al., 2013; Pathak et al., 2018), we argue that the *Fgf21* response to exercise in mice housed in TN conditions accurately reflects the human physiological response, although further research is needed for confirmation.

Irisin is expressed in various tissues, including skeletal muscle, adipose tissue and the brain (Fatouros, 2018). Research on the impact of exercise on irisin expression yields conflicting results. Human studies have reported varied responses, with acute exercise leading to an increase (Daskalopoulou et al., 2014; Huh et al., 2015; Lee et al., 2014; Otero‐Diaz et al., 2018), no change (Tsuchiya et al., 2014) or a decrease (Archundia‐Herrera et al., 2017; Bubak et al., 2017; Eaton et al., 2018; Kurdiova et al., 2014; Tsuchiya et al., 2014) in circulating irisin levels. Chronic exercise also showed contradictory outcomes for irisin levels (Bostrom et al., 2012; Fain et al., 2013; Lu et al., 2016; Norheim et al., 2014; Quinn et al., 2015; Rocha‐Rodrigues et al., 2016; Timmons et al., 2012; Wu et al., 2014). Irisin promotes the browning of white adipose tissue, as evidenced by experiments in animals conducted at RT (Bostrom et al., 2012; Cao et al., 2011; Knudsen et al., 2014; Peppler et al., 2018; Stanford et al., 2015; Trevellin et al., 2014; Wu et al., 2014). Wu et al. (2014) found that endurance exercise had no significant impact on muscle *Fndc5* (irisin) mRNA and circulating irisin but upregulated iWAT *Fndc5* (irisin) mRNA expression. They suggested that exercise promotes the browning of white adipose tissue through the upregulation of iWAT irisin rather than systemic irisin. This aligns with our study, in which we observed an increase in iWAT *Fndc5* (irisin) mRNA expression after acute exercise at RT. However, most human studies do not support the idea that exercise promotes the browning of white adipose tissue (Camera et al., 2010; Nakhuda et al., 2016; Pino et al., 2016; Tsiloulis et al., 2018; Zuriaga et al., 2017). Interestingly, the effect of exercise on promoting the browning of white adipose tissue significantly weakened in mice housed in TN conditions (Aldiss et al., 2020; McKie et al., 2019), although the specific molecular mechanisms remain unclear. Our study found that acute exercise can upregulate iWAT *Fndc5* (irisin) mRNA expression in mice housed at RT, but not in those housed at TN. We argue that exercise failed to induce the browning of white adipose tissue, which might be explained by the inability of exercise to upregulate iWAT *Fndc5* (irisin) mRNA expression in mice housed at TN. However, further research is needed to confirm this hypothesis. Based on these findings, we argue that regarding the irisin response to exercise, mice housed in TN conditions are representative of the real‐life response in humans.

Advances in multiomics have revealed many exerkines associated with fitness, insulin sensitivity and metabolic outcomes (Contrepois et al., 2020; Diaz‐Canestro et al., 2023; Robbins & Gerszten, 2023). Although promising for research and drug development, these findings pose challenges (Gubert & Hannan, 2021). Changes in exerkines vary with the exercise type, intensity and duration, necessitating standardized research approaches. The regulatory network of exerkines and their role in inter‐organ communication also require further exploration. Moreover, there is significant individual variability in exerkine responses to exercise, which might explain ‘exercise resistance’ (Bell et al., 2022; Diaz‐Canestro et al., 2023). Our study adds that housing temperature significantly influences exerkine expression. Long‐term exercise in TN conditions alters exerkines such as like Fgf21, Gdf15, fetuin‐A and irisin in a different way compared with RT conditions, indicating temperature‐sensitive mediation of the benefits of exercise. This insight is vital for developing effective exercise‐mimicking drugs that replicate the interplay between exercise and environmental conditions.

In this study, we found that in the TN environment, chronic exercise significantly improved glucose tolerance in mice, probably owing to the combined effects of various metabolic regulatory proteins and exerkines. Notably, exercise in the TN environment significantly reduced the p‐GS/t‐GS ratio, indicating an increase in GS activity in muscle, which might enhance glucose uptake and storage. Additionally, the decrease in hepatic levels of fetuin‐A and Gdf15, along with an increase in apelin, might reduce insulin resistance and further support improved glucose metabolism. In skeletal muscle, the upregulation of Metrnl and downregulation of Fgf21 suggest enhanced glucose metabolic activity, while the upregulation of Fgf21 in adipose tissue supports increased lipolysis and insulin sensitivity. In contrast, exercise in the RT environment did not significantly affect glucose tolerance, which might be related to a different molecular response profile; for instance, the downregulation of irisin in muscle and decreases in spexin and Fgf21 in adipose tissue potentially limit improvements in fat and glucose metabolism.

The limitations of this study should be acknowledged. First, we did not perform exercise phenotyping, hence detailed assessments of exercise capacity and adaptability were not conducted. Second, the number of replicates was limited to three (*n* = 3), which might affect the robustness and reproducibility of our findings. Third, tissue‐specific measurements of glucose uptake and insulin sensitivity were not included, although such assessments could provide further insights into the mechanisms of glucose tolerance. Fourth, we did not measure core body temperature during exercise, which might have introduced confounding effects, particularly in the TN environment, where overheating could potentially influence the results. Finally, whole‐body measurements of energy expenditure, such as indirect calorimetry, were not performed. These measurements would have provided valuable insights into the effects of exercise and environmental temperature on metabolic rate and overall energy balance, further elucidating the systemic effects observed in this study.

## 5 CONCLUSION

This study underscores the importance of considering housing temperature in exercise research. The findings highlight that housing temperature significantly affects the metabolic benefits of exercise and exerkine expression. Future research should adopt TN conditions to minimize confounding effects and accurately assess the metabolic benefits of exercise. Understanding the interplay between exercise, housing temperature and metabolic regulation will enhance the development of targeted interventions for improving metabolic health.

## AUTHOR CONTRIBUTIONS

Rao Zhijian: Conceptualization; Writing—Original draft preparation; Funding acquisition. Geng Xue: Methodology; Data curation; Data interpretation. Huang Peng: Methodology; Data acquisition Visualization; Investigation. Wei Qiangman: Software; Data analysis; Validation. Liu Shijie: Methodology; Data acquisition; Validation. Qu Chaoyi: Writing—Reviewing and Editing; Revising the manuscript. Zhaojiexiu: Conceptualization; Supervision; Funding acquisition. All authors have read and approved the final version of this manuscript and agree to be accountable for all aspects of the work in ensuring that questions related to the accuracy or integrity of any part of the work are appropriately investigated and resolved. All persons designated as authors qualify for authorship, and all those who qualify for authorship are listed.

## CONFLICT OF INTEREST

The authors declare no conflicts of interest.

## Data Availability

All data supporting the findings of this study are included within the manuscript. No additional data are available.
